# Role of Bile Acids in Dysbiosis and Treatment of Nonalcoholic Fatty Liver Disease

**DOI:** 10.1155/2019/7659509

**Published:** 2019-06-24

**Authors:** Caihua Wang, Chunpeng Zhu, Liming Shao, Jun Ye, Yimin Shen, Yuezhong Ren

**Affiliations:** ^1^Department of Gastroenterology, The Second Affiliated Hospital, College of Medicine, Zhejiang University, Hangzhou 310009, China; ^2^Department of Endocrinology and Metabolism, The Second Affiliated Hospital, College of Medicine, Zhejiang University, Hangzhou 310009, China

## Abstract

Nonalcoholic fatty liver disease (NAFLD) is a major health threat around the world and is characterized by dysbiosis. Primary bile acids are synthesized in the liver and converted into secondary bile acids by gut microbiota. Recent studies support the role of bile acids in modulating dysbiosis and NAFLD, while the mechanisms are not well elucidated. Dysbiosis may alter the size and the composition of the bile acid pool, resulting in reduced signaling of bile acid receptors such as farnesoid X receptor (FXR) and Takeda G protein-coupled receptor 5 (TGR5). These receptors are essential in lipid and glucose metabolism, and impaired bile acid signaling may cause NAFLD. Bile acids also reciprocally regulate the gut microbiota directly via antibacterial activity and indirectly via FXR. Therefore, bile acid signaling is closely linked to dysbiosis and NAFLD. During the past decade, stimulation of bile acid receptors with their agonists has been extensively explored for the treatment of NAFLD in both animal models and clinical trials. Early evidence has suggested the potential of bile acid receptor agonists in NAFLD management, but their long-term safety and effectiveness need further clarification.

## 1. Introduction

Nonalcoholic fatty liver disease (NAFLD) is emerging as the leading cause of chronic liver disease worldwide. It is diagnosed when fat is more than 5% of the weight of the liver. It affects 10-40% of the adult population worldwide and 30-40% of adults in the US [1]. Researchers have also found that 40-80% of type 2 diabetic patients and 30-90% of the obese population have NFALD [2]. NAFLD encompasses a spectrum of liver pathologies, ranging from simple fatty liver (steatosis) to nonalcoholic steatohepatitis (NASH), cirrhosis, and hepatocellular carcinoma in patients without a history of alcoholism. It can affect people of any age and any ethnicity. However, Hispanics and Asians are at higher risk for the disease [3]. It is estimated that approximately 20% of patients in the US with NAFLD have NASH [4]. A subset of NAFLD patients will evolve to cirrhosis and hepatocellular carcinoma. In addition to liver-related morbidity and mortality, NAFLD has been independently associated with extrahepatic diseases such as type 2 diabetes mellitus, cardiovascular diseases, and chronic kidney diseases [5]. Besides, NAFLD may add increased risk for extrahepatic cancers, especially in the gastrointestinal tract [6]. Furthermore, NAFLD has been closely associated with dysbiosis, the imbalance in the gut microbiota [7].

It has been estimated that human gut microbiota is composed of 300-500 species with a total of 10^13^ to 10^14^ bacteria. The collective genome (“microbiome”) encodes at least 100 times as many genes as the human genome. The stomach and small intestine have only a few species of bacteria, while the large intestine contains high densities and varieties of living bacteria, reaching concentrations of up to 10^11^ or 10^12^ cells/g of luminal tissues [8]. In a healthy human, host and bacterial colonizers maintain a homeostasis that benefits both parties. The host provides an environment and source of nutrients for the microbiota. On the other hand, the gut microbiota is crucial for the synthesis of essential amino acids and vitamins and the breakdown of indigestible components in the diet such as plant polysaccharides [9]. In general, dysbiosis can be categorized into three types: loss of beneficial bacteria, the expansion of harmful bacteria, and a general loss of diversity of bacteria [10]. The three types of dysbiosis are not mutually exclusive and can occur simultaneously in the same host. In addition to NAFLD, dysbiosis has been implicated in a plethora of diseases including inflammatory bowel disease, metabolic disorders, obesity, type 2 diabetes, and colorectal cancer. During the past decade, many studies support that dysbiosis and NAFLD are closely related to malfunction in bile acid signaling [11].

Bile acids are essential molecules in fat absorption [12]. Bile acids are produced by hepatocytes via oxidation of cholesterol in a multistep process to form the primary bile acids, such as cholic acid and chenodeoxycholic acid. They are synthesized via the classical pathway and the alternative pathway, with the classical pathway accounting for at least 75% of the products [13]. In hepatocytes, primary bile acids are further conjugated to glycine or taurine to form conjugated bile acids. Conjugating bile acids have pKa values between 1 and 4 which make them water-soluble when entering into the duodenum with a pH between 3 and 5 and allow them to perform their physiologic function of emulsifying and solubilizing fats [14]. Bile acids are secreted into the bile canaliculi via the bile salt export pump. Then, bile with the newly formed bile acids is transported through a system of the biliary tree to the gallbladder, where it is stored and concentrated during an empty stomach. Upon food consumption, the bile is released into the duodenum following gallbladder contraction in response to cholecystokinin produced by enteroendocrine I cells [15]. The intestinal microbiota converts primary bile acids into secondary bile acids such as deoxycholic acid, lithocholic acid, and ursodeoxycholic acid [16]. Approximately 95% of bile acids are actively reabsorbed in the distal ileum by the enterocytes and transported to the liver through the portal vein, while the remaining 5% is deconjugated by the gut microbiota and excreted in the feces. However, a small fraction of bile acids escapes hepatic uptake, flows into the systemic circulation, and reaches the peripheral tissues where they exert peripheral effects [15].

In addition to their role in fat absorption, bile acids are also signaling molecules and regulate glucose and lipid metabolism through activation of the farnesoid X receptor (FXR), a multipurpose nuclear receptor, and the Takeda G protein-coupled receptor 5 (TGR5). Impaired bile acid signaling was reported in patients with NAFLD and dysbiosis [17]. On the other hand, improved bile acid signaling via bile diversion altered the profile of gut microbiota and regulated glucose and lipid homeostasis, resulting in improved metabolic phenotype [18]. The goal of this review article is to summarize the existing literature on the role of bile acid signaling in dysbiosis and pathogenesis of NAFLD, as well as the application of bile acid receptor agonists in the treatment of NAFLD from animal models to clinical trials.

## 2. Dysbiosis

Despite the wide microbial diversity between adults, Bacteroidetes, Firmicutes, Proteobacteria, and Actinobacteria are the only 4 predominant phyla in the gut microbiota, with Bacteroidetes and Firmicutes occupying more than 95% of the phylogenetic types [19]. Some Firmicutes are butyrate producers in the colon and essential in colonic health in humans [20]. In contrast, Bacteroidetes have large numbers of genes encoding for carbohydrate active enzymes such as eliminases, glycosyltransferases, and glycoside hydrolases. This allows them to utilize a variety of energy sources in the gut depending on availability, by selective mechanisms to control gene expression [21]. Bacteroidetes are also the phylum with the greatest number of producers for B group vitamins [22]. On the other hand, *Escherichia coli* (*E. coli*), a species of bacteria from the family Enterobacteriaceae which belongs to the phylum *Proteobacteria*, can generate vitamin K2 to benefit the host [23].

Dysbiosis is defined as microbial imbalance or maladaptation on or inside the body. By comparing distal gut microbiota of genetically obese and lean mice, it has been demonstrated that obesity is associated with a decrease in Bacteroidetes and an increase in Firmicutes, resulting in the increased capacity to harvest energy from the diet. Colonization of germ-free mice with microbiota from genetically obese mice led to a significant increase in body fat than that from lean mice [24]. Obese mice induced by a fat-rich Western diet had a lower Bacteroidetes/Firmicutes ratio in distal gut microbiota [25]. Similarly, obese people displayed a decreased ratio of Bacteroidetes/Firmicutes, which was reverted after diet [26]. Additionally, obese rats induced by a fat-rich Western diet had the expansion of *Bilophila wadsworthia* in the intestine. This bacterial bloom promoted hepatic conjugation of bile acids, from glycocholic to taurocholic acid, resulting in solubilizing the hydrophobic fat in the diet and delivery of taurine-derived sulfur to the distal bowel [27].

## 3. Reciprocal Regulation between Bile Acids and Dysbiosis

Intestinal microbiota is known to regulate bile acid homeostasis via biotransformation such as deconjugation, dehydroxylation, oxidation, and desulfation. In terms of deconjugation, bacterial genera such as *Bacteroides*, *Lactobacillus*, *Bifidobacterium*, *Clostridium*, and *Listeria* produce bile salt hydrolases (BSHs), which deconjugate taurine and glycine groups in the primary bile acids produced in the liver [16]. In germ-free or antibiotic-treated rats, the deconjugation of taurine by gut bacteria was blocked, shifting the balance to almost exclusively taurine-conjugated bile acids, which resulted in increased taurine-conjugated bile acids in the liver, heart, and kidney [28]. Mice colonized with BSH-deleted *Bacteroides thetaiotaomicron* displayed a high level of taurine-conjugated bile acids and altered metabolism including decreased weight gain and respiratory exchange ratios, as well as changes in immune pathways in the gut and liver [29]. For dehydroxylation, *Clostridium* and *Eubacterium* of the Firmicutes phylum generate bile acid 7*α*-dehydroxylase, which converts primary bile acids (cholic acid and chenodeoxycholic acid) into secondary bile acids (deoxycholic acid, lithocholic acid, and ursodeoxycholic acid) [30]. The 7*α*-dehydroxylation occurs after deconjugation and is the most physiologically significant conversion of bile acids in humans. In a gnotobiotic mouse model, intestinal bile acid 7*α*-dehydroxylation by *Clostridium scindens* has been associated with protection again *Clostridium difficile* infection [31]. With regard to oxidation, the bacterial genera such as *Bacteroides*, *Clostridium*, *Eubacterium*, *Escherichia*, *Eggerthella*, *Peptostreptococcus*, and *Ruminococcus* generate bile acid hydroxysteroid dehydrogenases (HSDHs) which convert toxic bile acids into ursodeoxycholic acid, which is less toxic to human cells and more water-soluble [32]. Finally, several gut bacteria such as *Clostridium* sp. Strain S2 produce sulfatases which are able to increase the desulfation of bile acids [33]. Desulfation of bile acids by gut bacteria facilitates bile acid reabsorption [34] and is essential for the homeostasis of the bile acid pool [34].

On the other hand, bile acids are the most significant endogenous metabolites correlating with dysbiosis [35]. When taurocholic acid, but not glycocholic acid, was added to mice fed with a low-fat diet, it promoted the expansion of *Bilophila wadsworthia* and resulted in colitis in genetically susceptible IL-10 knockout mice [36]. In patients with cirrhosis, the chenodeoxycholic level was positively correlated with the abundance of fecal *Enterobacteriaceae* while the deoxycholic acid level was positively correlated with *Ruminococcaceae* [37]. Islam et al. reported that when rats were fed with diets supplemented with cholic acid, they induced phylum-level alterations in the composition of the gut microbiota with a decrease in Bacteroidetes and an increase in Firmicutes similar to those induced by high-fat diets [38]. Clements et al. showed that biliary obstruction, which reduced the level of intraluminal bile acids, increased the Gram-negative aerobic population and induced bacterial translocation in the intestine [39]. On the other hand, administration of conjugated bile acids to cirrhotic rats elevated bile acid secretion, inhibited intestinal bacterial overgrowth, and reduced bacterial translocation [40].

Bile acids have both direct influences on gut microbiota and indirect influences mediated by FXR. It has been demonstrated that deoxycholic acid is an order of magnitude greater than cholic acid in antibacterial activity [16, 41]. In addition, unconjugated bile acids possess more potent antibacterial action than conjugated counterparts via membrane disruption and leakage of cellular contents [35]. Sung et al. demonstrated that hydrophobic bile acids (taurodeoxycholic acid and deoxycholic acid) had more significant inhibition on the growth of bacteria when compared with the hydrophilic bile acids (taurocholic acid, chenodeoxycholic acid, and tauroursodeoxycholic acid) [42]. Moreover, bile acids may induce the expression of stress response genes in gut bacteria in response to membrane perturbation, oxidative stress, and DNA damage [43]. In terms of FXR involvement, Inagaki et al. reported that FXR agonist GW4064 inhibited bacterial overgrowth and mucosal injury in the small intestine caused by biliary obstruction by inducing the expression of *Ang1*, *iNos*, and IL-18 [44]. Zheng et al. showed that a high-fat diet elevated intestinal bile acid pool within 12 h, followed by a change in gut microbiota at 24 h, indicating that bile acids might regulate the composition of gut microbiota. Feeding mice with a normal diet supplemented with taurocholic acid and glycocholic acid also expanded the bile acid pool, especially for secondary bile acids, and increased the abundance of *Verrucomicrobia* compared with the normal diet. However, the use of the FXR agonist GW4064 to inhibit bile acid biosynthesis prevented the development of the obese phenotype and the alteration of the gut microbiota, demonstrating that regulation of gut microbiota by bile acids is FXR dependent [45]. Bile acid receptor FXR knockout mice on a high-fat diet had high levels of primary bile acids such as beta-muricholic acid and tauro-beta-muricholic acid. The mice had elevated relative abundance of Firmicutes and decreased relative abundance of Bacteroidetes compared with wild-type mice on the same diet [46]. Furthermore, Pathak et al. demonstrated that the FXR agonist fexaramine induced lithocholic acid-producing bacteria *Acetatifactor* and *Bacteroides*, increased taurolithocholic acid, and elevated glucose tolerance [47].

## 4. Bile Acid Signaling

Bile acids bind and activate FXR in the order of chenodeoxycholic acid > lithocholic acid > deoxycholic acid > cholic acid. On the other hand, the most efficacious bile acid ligands for TGR5 are in the order of lithocholic acid > deoxycholic acid > chenodeoxycholic acid > cholic acid [48]. Bile acids are modulators for lipid and glucose metabolism which is mediated mainly by FXR and TGR5 [49]. Bile acids reduce triglyceride levels via a pathway involving FXR and the small heterodimer partner (SHP), as well as sterol regulatory element-binding protein 1 (SREBP-1), which targets lipogenic genes [50]. Activation of FXR also promotes the expression of the peroxisome proliferator-activated receptor alpha (PPARalpha), which is a nuclear receptor that regulates lipid metabolism, glucose homeostasis, and anti-inflammatory activities [51]. Cariou et al. reported that mice with FXR gene knockout had impaired glucose tolerance and insulin resistance. The mice also displayed reduced adipose tissue mass and serum leptin levels as well as increased plasma-free fatty acid levels. The FXR agonist GW4064 promoted insulin signaling and insulin-stimulated glucose uptake in adipocytes. Furthermore, GW4064 treatment increased insulin resistance in genetically obese ob/ob mice *in vivo* [52]. In another study with FXR knockout mice, mice had increased free fatty acid levels and developed severe NAFLD [53]. Zhang et al. found that the FXR agonist GW4064 or hepatic overexpression of the FXR gene reduced blood glucose levels in both genetically diabetic db/db and wild-type mice. Activation of FXR in db/db mice increased hepatic glycogen synthesis and the glycogen level via enhancing insulin sensitivity [54]. On the other hand, bile acid signaling may also enhance insulin sensitivity and reduce obesity via activating TGR5. Brighton et al. reported that bile acids were bound with TGR5 on the basolateral L cell membrane and stimulated the release of glucagon-like peptide-1 (GLP-1), which is capable of increasing insulin release, lowering glucagon secretion from the pancreas, and reducing food intake and gastrointestinal motility [55]. Thomas et al. demonstrated that TGR5 overexpression enhanced GLP-1 release in vivo, which increased glucose tolerance and improved liver and pancreatic function. In addition, the TGR5 agonist induced GLP-1 release via elevating the intracellular ATP/ADP ratio and intracellular calcium mobilization in enteroendocrine L cells [56].

Bile acid signaling also controls bile acid levels. The physiological concentration of bile acids is regulated through FXR and TGR5, by inhibiting the expression of cytochrome P450 7A1 (CYP7A1), a rate-limiting enzyme in bile acid synthesis [57]. In the liver, FXR promotes the expression of SHP, an atypical nuclear receptor lacking a DNA binding domain. Then, SHP-1 inhibits the activity of liver receptor homolog 1 (LRH-1), an orphan nuclear receptor that positively regulates CYP7A1 expression via binding to a response element in the CYP7A1 promoter [57]. In the small intestine, FXR regulates bile acid levels via FGF15 in the mouse (FGF19 in human)/fibroblast growth factor receptor 4 (FGFR4)/ERK1/2 pathway, which inhibits bile acid synthesis by repressing transcription of CYP7A1 and prevents the accumulation of toxic bile acids in the liver [58]. Mice lacking FGF15 had increased hepatic CYP7A1 enzyme activity and fecal bile acid excretion [59]. Lou et al. found that TGR5 activation induced the expression of proinflammatory cytokines in Kupffer cells and suppressed the expression of CYP7A1 in murine hepatocytes [60]. These regulatory mechanisms contribute to the delicate bile acid feedback regulation.

## 5. Evidences That Dysbiosis Links NAFLD

Wigg et al. were the first to report the association between dysbiosis and NAFLD. They found that NAFLD patients had bacterial overgrowth in the small intestine and higher TNF-*α* levels compared with control subjects [61]. In a comparison study of intestinal microbiota among adults with biopsy-proven NAFLD and living liver donors as healthy controls, quantitative real-time polymerase chain reaction analysis of stool samples revealed that NAFLD patients had a lower percentage of Bacteroidetes compared to health controls [62]. Wong et al. reported that NAFLD patients displayed a decrease in *Faecalibacterium* (Firmicutes) and *Anaerosporobacter* (Firmicutes) but an increase in *Parabacteroides* (Bacteroidetes) and *Allisonella* (Firmicutes) in gut microbiota [63]. In obese patients with NAFLD, deep sequencing of the fecal microbiota showed overrepresentation of *Lactobacillus* and *Lachnospiraceae* and underrepresentation of *Ruminococcaceae* in the phylum Firmicutes [64]. Zhu et al. examined the composition of gut bacteria of NAFLD, obese, and healthy children by 16S ribosomal RNA pyrosequencing. The representation of *Escherichia* with alcohol-producing capacity was similar between healthy and obese groups but was significantly increased in the NAFLD group. In addition, there was an increase in blood-ethanol concentration in children with NAFLD [65]. In another study of pediatric NAFLD patients, NAFLD progression was accompanied by an increases in *Ruminococcus* (Firmicutes) and *Dorea* (Firmicutes) in gut microbiota, indicating an association between gut microbiota and disease severity [66].

Nevertheless, dysbiosis has also been related to metabolic diseases, since several studies supported that increased Firmicutes and decreased Bacteroidetes may lead to obesity [24, 67]. In a prospective cross-sectional study examining the gut microbiota composition of 39 adults with biopsy-proven NAFLD, Da Silva et al. found that NAFLD patients had a lower abundance of *Ruminococcus*, *F. prausnitzii*, and *Coprococcus*. Furthermore, this lower abundance of gut microbiota was independent of obesity and insulin resistance [68]. Le Roy et al. reported that when wild-type mice were fed with a high-fat diet, only mice that received fecal transplantation from NAFLD mice developed the disease [69]. Mouzaki et al. found that Bacteroidetes were less abundant in NAFLD patients compared with the control, after adjusting for body mass index and weight-adjusted calorie intake [70]. These findings reveal that dysbiosis contributes to the development of NAFLD independent of metabolic abnormality.

## 6. Mechanisms Where Dysbiosis Is Involved in NAFLD

Dysbiosis plays an essential part in the development of NAFLD. Proposed mechanisms in the relationship of dysbiosis and NAFLD include augmented capacity for energy extraction from food, increased intestinal permeability, elevated serum LPS, overgrowth of bacteria, decreased choline metabolism, and suppressed bile acid signaling. The following paragraphs will delineate the proposed mechanisms one by one.

One mechanism by which dysbiosis promotes NAFLD is an increased energy yield from food. ob/ob mice as well as obese humans have an altered composition of their gut microbiota and increased short-chain fatty acids [24]. Short-chain fatty acids contain 1–6 carbons and are the main product of bacterial fermentation of undigested dietary fiber, thus improving energy extraction from the diet [71]. Loss of Gpr41, a G protein-coupled receptor for short-chain fatty acids, is associated with reduced efficiency of energy harvest from the diet [72].

Secondly, elevated intestinal permeability is closely associated with NAFLD. The intestinal epithelial cells, a physical barrier between potential pathogens and the underlying lamina propria, are linked together through tight junctions and crucial for maintaining intestinal immune homeostasis. Dysbiosis can target various signal transduction pathways, regulate the expression and distribution of tight junction proteins, and increase intestinal permeability [73]. Elevated permeability leads to translocation of bacterial components that triggers the inflammatory response of the liver through TLR4 (receptor for LPS), TLR5 (receptor for bacterial flagellin), and TLR9 (receptor for bacterial DNA) [74]. In patients with NAFLD, both intestinal permeability and bacterial growth were increased and correlated with the severity of steatosis [75]. ob/ob mice had reduced mucosal barrier function, modified distribution of tight junction proteins, and higher levels of proinflammatory cytokines compared with lean control mice [76].

Thirdly, LPS is endotoxin derived from the outer membrane of Gram-negative bacteria. LPS is recognized by TLR4 and triggers inflammatory response through the TLR4/NF-*κ*B signaling pathway [77]. Normally, only a small amount of LPS crosses the epithelial membrane, enters the portal system, and is degraded in the liver. In mice, dysbiosis induced by high-fat feeding increased plasma LPS levels, which activated TLR4 on Kupffer cells and hepatocytes, and initiated obesity and insulin resistance. In addition, LPS infusion in normal diet-fed mice produced a metabolic response similar to high-fat feeding [78]. In another study, inflammasome-deficiency mice developed NAFLD and were associated with increased levels of LPS in the portal vein and TNF-*α* expression. Cohoused wild-type mice with inflammasome-deficient mice developed NAFLD [79]. In a human study, Kapil et al. documented that patients with NAFLD had significantly higher LPS and TLR4 levels compared with patients without NAFLD [80]. In patients with NAFLD, Vespasiani-Gentilucci et al. found that the TLR4 expression was significantly elevated and was associated with the activation of fibrogenic cells and the degree of fibrosis [81]. In a prospective cohort study of 920 patients, Wong et al. reported that the levels of LPS-binding protein were significantly increased and were associated with the development of NAFLD [82].

Fourthly, many studies have also shown that small intestinal bacterial overgrowth (SIBO) is associated with NAFLD. In a study of 372 eligible patients who were tested for SIBO and had liver imaging, 141 (37.9%) were tested positive for SIBO and 231 (62.1%) were negative. NAFLD occurred in 45.4% (64/141) of the SIBO-positive group, while only 17.3% (40/231) in the negative group [83]. Kapil et al. reported that 12 out of 32 patients with NAFLD had SIBO with *Escherichia coli* as the predominant bacterium. Patients with SIBO had significantly higher levels of LPS, CD14 mRNA, NF-*κ*B mRNA, and TLR4 protein compared with those without SIBO [80]. Sabate et al. documented that the frequency of positive SIBO was higher in obese patients (24/136, 17.1%) than in healthy subjects (1/40, 2.5%). In a multivariate analysis, SIBO was an independent factor of NAFLD [84]. Shanab et al. found that NAFLD patients had a higher prevalence of SIBO (77.78% vs. 31.25%), which was associated with the enhanced expression of TLR4 and IL-8 [85]. In a study of 125 obese children, 47 (37.6%) had intestinal dysbiosis and 47 (37.6%) were SIBO positive compared with 3.3% positive in the controls. NAFLD was present in 28/47 (59.5%) of the SIBO-positive obese group, compared with only 8/78 (10.2%) of the SIBO-negative obese group and 0/120 (0%) of the controls. Obese children with SIBO positivity had higher rates of abnormality in liver function [86].

Fifthly, choline is an essential nutrient and a main methyl donor in the physiological processes. Mice with a methionine- and choline-deficient diet have been shown to develop NAFLD with inflammasome activation, IL-1*β* secretion, and liver inflammation [87]. Phosphatidylcholine, an important choline metabolite in the liver, is required for VLDL secretion, which is a mechanism to dispose excessive fat accumulation in the liver [88]. In patients requiring long-term parenteral nutrition, choline deficiency was reported to cause NAFLD, which was reversed with choline supplementation [89]. Deletion of betaine-homocysteine S-methyltransferase in mice reduced choline levels in the tissue, resulting in fatty liver and hepatocellular carcinomas [90]. In a study of 15 female subjects on well-controlled diets, Spencer et al. found that choline deficiency resulted in the development of fatty liver. Additionally, *Gammaproteobacteria* was negatively correlated with fat accumulation in the liver, while *Erysipelotrichi* had a positive correlation [91]. Mouse strain 129S6 with a high-fat diet, known to be susceptible to NAFLD, had a low plasma level of phosphatidylcholine and high urinary excretion of methylamines. Conversion of choline into methylamines by microbiota in strain 129S6 reduced the bioavailability of choline and mimicked the effect of choline-deficient diets [92].

Finally, impaired bile acid signaling is probably the most important mechanism for NAFLD development. By binding to the FXR and TGR5, bile acids were reported to increase insulin sensitivity and decrease hepatic gluconeogenesis and circulating triglycerides [93]. Yang et al. found that patients with NAFLD had lower levels of hepatic FXR and elevated levels of SREBP-1C and triglyceride, while there was no change in SHP expression [94]. Dasarathy et al. reported that patients with NAFLD had elevated fasting plasma bile acids [95]. In patients with NAFLD, Jiao et al. reported that serum levels of primary and secondary bile acids were elevated. Gut microbiota had increased abundance in *Escherichia* and *Bilophila*, which metabolize taurine and glycine, indicating increased production of secondary bile acids. Proportion for deoxycholic acid (secondary) was increased, while the proportion for chenodeoxycholic acid (primary) was decreased in the bile acid pool. The expression of SHP was not changed in the livers, while the downstream effector gene CYP7A1 was elevated. Furthermore, serum FGF19 levels were decreased, indicating impaired FXR signaling in NAFLD [17]. Mouzaki et al. found that bile aid synthesis was higher in patients with NAFLD compared with the control. Bacteroidetes and *Clostridium leptum* counts were less abundant in NAFLD patients compared with the control, after adjusting for body mass index and weight-adjusted calorie intake [70]. FXR is also responsible for the beneficial effects of bariatric surgery, which is associated with improvements in type-2 diabetes and NAFLD. In mice with diet-induced obesity, bariatric surgery elevated circulating bile acids and altered gut microbiota. In the absence of bile acid signaling (FXR knockout mice), the effectiveness of bariatric surgery in weight loss and elevated glucose tolerance was significantly decreased [96]. Parseus et al. reported that FXR-deficient mice had a significantly higher proportion of tauro-beta-muricholic acid compared with wild-type mice, suggesting reduced capacity to deconjugate bile acids. FXR-deficient mice showed mild NAFLD even in the presence of gut microbiota, suggesting that dysbiosis-induced NAFLD is FXR dependent [46]. Of note, the adverse reactions of FXR agonists were also observed in a clinical trial. The major adverse events were pruritus, which was found in 23% of the OCA-treatment group and increased the serum low-density lipoprotein cholesterol level in the clinical trial [97].

## 7. Bile Acid Receptor Agonists for NAFLD in Animal Studies

Many studies have been conducted so far to study the effectiveness of bile acid receptor agonists in animal models of NAFLD (Table 1). In a mouse model of NAFLD, McMahan et al. reported that treatment of genetically obese db/db mice with dual bile acid FXR/TGR5 receptor agonist INT-767 reduced hepatic expression of profibrotic/proinflammatory genes and ameliorated the histological features of NAFLD. The treatment increased the anti-inflammatory Ly6C (low) phenotype of intrahepatic monocytes and elevated M2 phenotype of intrahepatic macrophages. Furthermore, treatment of monocytes with INT-767 in vitro increased the anti-inflammatory IL-10 level through a cAMP-dependent pathway [98]. Roth et al. compared the effectiveness of INT-767 in a diet-induced ob/ob mouse model of NAFLD with FXR agonist obeticholic acid (OCA). They found that INT-767 exerted greater therapeutic potency and efficacy than OCA in improving NAFLD histology [99]. To elucidate the relative importance of FXR versus TGR5 in mediating the effects of INT-767, Jadhav et al. used mice with FXR, TGR5, or SHP gene knockout to study NAFLD development. They found that INT-767 reversed obesity, hypercholesterolemia, and NAFLD by activation of FXR and/or TGR5. The results indicated that dual activation of FXR and TGR5 is an attractive strategy for treatment of NAFLD and metabolic disorders [100]. In a rat model of NAFLD, Hu et al. found that INT-767 treatment significantly reduced hepatic lipid accumulation and infiltration of immune cells. INT-767 also corrected abnormal lipid metabolism and insulin resistance. Furthermore, levels for endotoxin, TNF-*α*, and NF-*κ*B were significantly reduced after INT-767 treatment [101]. In a rabbit model of high-fat diet-induced NAFLD and metabolic syndrome, Comeglio et al. found that long-term treatment with INT-767 ameliorated visceral adipose tissue accumulation, hypercholesterolemia, and NAFLD. INT-767 also alleviated insulin resistance and induced mitochondrial function, resulting in preadipocyte differentiation toward a metabolically healthy phenotype [102].

Ma et al. discovered that treatment with the FXR agonist GW4064 reduced weight gain in mice fed with a high-fat diet. GW4064 significantly lowered triglyceride and free fatty acid levels in the liver and attenuated NAFLD. GW4064 treatment also alleviated hepatic inflammation while having no effect on white adipose tissue. In addition, GW4064 decreased hyperinsulinemia and hyperglycemia through reducing mRNA levels of phosphoenolpyruvate carboxykinase and glucose-6-phosphatase, two key enzymes in gluconeogenesis [103]. de Oliveira et al. evaluated the effectiveness of selective bile acid receptor agonists INT747 (FXR) and INT777 (TGR5) to treat metabolic abnormality in mice induced by ovariectomy and high-fat diet. INT747 or INT777 treatment attenuated bodyweight gain and increased energy expenditure. Both treatments alleviated liver steatosis and normalized liver triglyceride and cholesterol content. Gene expression for liver acyl-CoA oxidase and carnitine palmitoyltransferase 1*α* was induced after INT747 and INT777 treatment, indicating increased fatty acid oxidation [104]. Haczeyni et al. showed that OCA improved adipose morphology, inflammation, and NAFLD in dietary but not metabolic obesity in mice [105]. Liles et al. demonstrated that the FXR agonist GS-9674 reduced serum transaminases, liver fibrosis, and steatosis in high-fat, cholesterol, and sugar diet-induced mouse NAFLD [106]. In another mice model of NAFLD induced by a high-fat diet, Zheng et al. discovered that treatment with altenusin (2076A), a natural FXR agonist, reduced fat mass and glucose level. Altenusin treatment also reversed hepatic lipid droplet accumulation and steatosis. These effects were lost in FXR knockout mice. Mechanistically, increased insulin sensitivity and suppression of genes for hepatic gluconeogenesis and lipogenesis may account for the metabolic benefits of altenusin [107]. Zhang et al. examined the effects of WAY-362450 (W450), a highly selective and potent FXR agonist, on a mouse model of NAFLD induced by a methionine- and choline-deficient diet. WAY-362450 treatment decreased enzymes of liver damage, infiltration of inflammatory cells, and hepatic fibrosis, which was correlated with a reduction in hepatic fibrosis markers. The effect was FXR dependent since the protection was lost in FXR-deficient mice [108]. Carino et al. found that C57BL/6 mice were insulin resistant and developed NAFLD after 18 weeks on a high-fat diet. The NAFLD features included severe steatohepatitis and fibrosis, increased hepatic triacylglycerol and cholesterol, and abnormal expression of SREPB1c, FAS, ApoC2, PPAR*α* and PPAR*γ*, *α*-SMA, *α*1 collagen, and MCP1 mRNAs. Treatment with BAR502, a dual agonist for FXR and TGR5, alleviated liver steatosis, inflammation, fibrosis, and abnormalities of the mRNAs. Furthermore, BAR502 improved adipose function with browning of white fat tissue [109]. The same group also discovered that both wild-type and TGR5 knockout mice fed with a high-fat diet developed NAFLD. Treating NAFLD mice with BAR501, a selective TGR5 receptor agonist, ameliorated insulin resistance and histology of NAFLD, while the effects were absent in TGR5 knockout mice [110]. Jin et al. reported that avermectin analogues, which are existing antiparasitic drugs, were partial agonists for FXR and had therapeutic effects for NAFLD. In mice with a high-fat diet, avermectin analogues decreased hepatic lipid accumulation, lowered serum cholesterol and glucose levels, and restored insulin sensitivity in a FXR-dependent manner [111].

## 8. Bile Acid Receptor Agonists for NAFLD in Clinical Trials

At present, there are no approved effective pharmacologic agents available for the management of NAFLD. The current treatment options include lifestyle change, weight loss, and probiotics. In a previous clinical trial, it has been shown that ursodeoxycholic acid, a weak bile acid receptor agonist, was ineffective in alleviating the degree of steatosis, necroinflammation, or fibrosis in NAFLD patients compared with the placebo control [112].

During the past decade, several clinical trials have been conducted to test the safety and effectiveness of OCA, a FXR ligand with 100 times higher affinity than chenodeoxycholic acid, in the treatment of NAFLD (Table 2). In a double-blind, placebo-controlled phase 2 trial, treatment with OCA for 6 weeks in 41 patients with NAFLD and type 2 diabetes mellitus dose-dependently improved insulin sensitivity and decreased body weight compared with the placebo. The treatment also significantly reduced markers for liver fibrosis and enzymes for liver damage such as gamma-glutamyl transferase and alanine aminotransferase. In addition, serum levels of low-density lipoprotein and fibroblast growth factor 19 were significantly increased compared with the placebo. Furthermore, the treatment reduced endogenous bile acid levels and hepatic cholesterol 7*α*-hydroxylase activity, indicating FXR activation [113]. In a double-blind, randomized, placebo-controlled phase 3a trial, treatment with OCA for 72 weeks in 141 patients with NAFLD significantly improved the histological characteristics of NAFLD, including hepatic steatosis, inflammation, hepatocyte ballooning, and fibrosis [97]. However, the treatment was associated with side effects which include pruritus, an increase in low-density lipoprotein, and a decrease in high-density lipoprotein [97]. The side effects on lipoproteins are explained by the fact that OCA increases the expression of cholesteryl ester transfer protein, which transfers cholesterol from the high-density lipoprotein to the very low-density lipoprotein and low-density lipoprotein [114]. However, the changes in lipoproteins were not identified in animal studies since mice and rats lack the cholesteryl ester transfer protein [114, 115]. To further examine the effects of OCA on lipoproteins, a randomized, double-blind, placebo-controlled phase 2 study, evaluating the effects of OCA and atorvastatin treatment on lipoprotein metabolism in subjects with NAFLD (ClinicalTrials.gov identifier: NCT02633956), has recently been completed and the findings will be available in the near future. A phase 3 study to determine the safety and efficacy of OCA in NAFLD patients is recruiting subjects around the world (NCT02548351). The main goal of this study is to compare the effect of OCA with placebo on liver histology and clinical outcomes in NAFLD patients with liver fibrosis. Another phase 3 study examining the efficacy and safety of OCA in NAFLD patients with compensated cirrhosis is also currently underway (NCT03439254). The main goal of this trial is to determine whether OCA can improve liver histology in a patient with compensated cirrhosis as a result of NAFLD.

Several selective nonbile acid FXR agonists, hopefully without the adverse effects on lipoproteins, are being evaluated for the treatment of NAFLD (Table 2). Gilead recently presented the clinical trial data of GS-9674 in NAFLD at The Liver Meeting® 2018. In this study, patients were randomized to GS-9674 100 mg, GS-9674 30 mg, or placebo once daily for 24 weeks. Results showed that 38.9% of patients treated with GS-9674 100 mg (*p* = 0.011 vs. placebo), 14% treated with GS-9674 30 mg (*p* = 0.87), and 12.5% with placebo had a reduction in hepatic fat by at least 30 percent. There were also improvements in liver biochemistry tests and a reduction in bile acid synthesis in the 30 mg and 100 mg arms compared with the placebo (NCT02854605). A separate phase 2 study is examining GS-9674, selonsertib, and GS-0976 alone or in combination, in NAFLD patients with advanced fibrosis (NCT02781584). Two more FXR agonists, LMB763 (NCT02913105) and LJN452 (NCT02855164), are also under clinical investigation to test their effects on safety, tolerability, and liver inflammation in patients with NAFLD.

## 9. Conclusion

NAFLD is an underlying disease for cirrhosis and hepatocellular carcinoma and a risk factor for the development of other diseases such as metabolic syndrome, cardiovascular diseases, and type 2 diabetes. There is a growing body of literature documenting the role of dysbiosis in the pathogenesis of NAFLD. Dysbiosis also disrupts homeostasis of bile acids and affects bile acid pool size and composition [116]. Primary bile acids are converted to secondary bile acids via gut microbiota. In addition to facilitate the absorption of lipids and fat-soluble vitamins, bile acids are also signaling molecules in hepatic and extrahepatic tissues to modulate lipid and carbohydrate metabolism and maintain energy homeostasis. When bound to the FXR and TGR5 receptors, bile acids increase insulin sensitivity and reduce hepatic gluconeogenesis and circulating triglycerides [53]. On the other hand, bile acids possess both direct antimicrobial effects on gut microbiota via membrane disruption and leakage of cellular contents [35] and indirect inhibitory effects on bacterial overgrowth and mucosal injury via FXR [44]. There is a suppressed bile acid signaling despite elevated levels of primary and secondary bile acids in NAFLD [17]. Modulation of bile acid signaling via bile acid receptor agonists inhibits bile acid biosynthesis [117], alleviates metabolic syndromes [100], ameliorates dysbiosis [47], and improves the histology of NAFLD [99]. Although preclinical and clinical studies have demonstrated that bile acid receptor agonists such as OCA are promising strategies for NAFLD, there are still many challenges to overcome such as FXR/TGR5 selectivity, tissue selectivity, and unwanted metabolic side effects. Further clinical trials with larger sample sizes, long-term safety, and efficacy data generated from improvement in liver histology are warranted.

## Figures and Tables

**Table 1:** Bile acid receptor agonists for NAFLD in animal studies.

References	Model	Agent, mechanism, and duration	Findings
McMahan et al. [98]	Obese db/db mice with NAFLD	INT-767, dual FXR/TGR5 agonist, 6 weeks	(1) Improved the histological features of NAFLD (2) Increased the proportion of intrahepatic monocytes with the anti-inflammatory phenotype
Roth et al. [99]	ob/ob mice with NAFLD	INT-767, dual FXR/TGR5 agonist, 6 weeks	(1) Improved the histological features of NAFLD (2) Reduced liver fibrosis, inflammation, and hepatocyte lipid droplet area
Jadhav et al. [100]	TGR5, FXR, Apoe, and SHP knockout mice with diet-induced NAFLD	INT-767, dual FXR/TGR5 agonist, 12 weeks	(1) Reversed atherosclerosis and NAFLD (2) Reduced high-fat diet-induced obesity dependent on activation of both TGR5 and FXR
Hu et al. [101]	High-fat diet-induced rat NAFLD	INT-767, dual FXR/TGR5 agonist, 4 weeks	(1) Reduced lipid accumulation and hepatic infiltration of immune cells (2) Restored lipid and glucose metabolism
Comeglio et al. [102]	High-fat diet-induced rabbit metabolic syndrome	INT-767, dual FXR/TGR5 agonist, 12 weeks	(1) Alleviated NAFLD and fat alterations (2) Restored insulin sensitivity and induced preadipocytes toward a metabolically healthy phenotype
Ma et al. [103]	High-fat diet-induced mouse NAFLD	GW4064, FXR agonist, 6 weeks	(1) Ameliorated NAFLD and liver inflammation (2) Reversed diet-induced hyperinsulinemia and hyperglycemia
de Oliveira et al. [104]	Ovariectomized and high-fat diet-induced mouse NAFLD	OCA (FXR) and INT-777 (TGR5), 4 weeks	(1) Corrected NAFLD and metabolic changes (2) Elevated energy expenditure and expression of key metabolic genes
Haczeyni et al. [105]	Dietary and metabolic obesity mouse NAFLD	OCA, 24 weeks	Improved glucose tolerance and NAFLD in dietary but not metabolic obesity mouse
Liles et al. [106]	High-fat, cholesterol, and sugar diet-induced mouse NAFLD	GS-9674, FXR agonist, 90 days	(1) Improved the histological features of NAFLD (2) Decreased serum transaminases and liver fibrosis
Zheng et al. [107]	High-fat diet-induced mouse NAFLD	Altenusin, FXR agonist, 3 weeks	(1) Reversed NAFLD, alleviated dyslipidemia and insulin resistance, and reduced body weight and fat mass (2) No effect on FXR knockout mice
Zhang et al. [108]	MCD diet-induced mouse NAFLD	WAY-362450, FXR agonist, 4 weeks	(1) Reduced inflammatory cell infiltration and fibrosis (2) Protection was lost in FXR knockout mice
Carino et al. [109]	High-fat/high-fructose diet-induced NAFLD	BAR502, dual FXR/TGR5 agonist, 8 weeks	(1) Alleviated NAFLD, increased insulin sensitivity, and elevated circulating levels of HDL (2) Improved browning of white fat tissue
Carino et al. [110]	High-fat/high-fructose diet-induced NAFLD	BAR501, TGR5 agonist, 9 weeks	(1) Reversed insulin resistance and NAFLD (2) Promoted energy expenditure and the browning of white fat tissue. Lost effect in TGR5 knockout mice
Jin et al. [111]	High-fat diet-induced mouse NAFLD	Avermectin, FXR agonist, 14 days	(1) Ameliorated NAFLD, reduced glucose levels, and improved insulin sensitivity (2) Protection was FXR dependent

NAFLD: nonalcoholic fatty liver disease; db/db: diabetic/diabetic; ob/ob: obese/obese; FXR: farnesoid X receptor; TGR5: Takeda G protein-coupled receptor 5; SHP: small heterodimer partner; MCD: methionine- and choline-deficient; OCA: obeticholic acid.

**Table 2:** Clinical trials studying effects of bile acid receptor agonists in NAFLD.

References	Drug, dose, frequency, and duration	Finding or objective	Phase of trial	Status
NCT00501592, (Mudaliar et al. [113])	OCA; 25 mg, 50 mg, or placebo; once daily; 6 weeks	Finding: improved insulin sensitivity and reduced markers of liver inflammation and fibrosis in patients with T2DM and NAFLD	Phase 2	Completed
NCT01265498 (Neuschwander-Tetri et al. [97])	OCA, 25 mg or placebo, once daily, 72 weeks	Finding: improved liver histology in noncirrhotic NAFLD	Phase 3	Completed
NCT02633956	OCA; 5 mg, 10 mg, 25 mg, or placebo; once daily	Objective: effects of OCA and atorvastatin treatment on lipoprotein metabolism in NAFLD	Phase 2	Completed
NCT02548351	OCA; 10 mg, 25 mg, or placebo; once daily	Objective: histology and liver-related clinical outcomes in patients with noncirrhotic NAFLD with liver fibrosis	Phase 3	Ongoing
NCT03439254	OCA; 10 mg, 25 mg, or placebo; once daily	Objective: liver histology in adults with compensated cirrhosis due to NAFLD	Phase 3	Ongoing
NCT01999101	GS-9674 (Px-104), 5 mg, 28 days	Objective: analysis of clinical chemistry, hematology, and assessment of clinical signs and adverse events of Px-104 in adult NAFLD patients	Phase 2	Terminated
NCT02854605. Abstract in The Liver Meeting 2018	GS-9674; 30 mg, 100 mg, or placebo; once daily; 24 weeks	Finding: decline in hepatic fat, improvement in liver biochemistry tests, and reduction in bile acid synthesis	Phase 2	Completed
NCT02781584	GS-9674, 30 mg, once daily, 12 weeks	Objective: treatment with GS-9674, selonsertib, and GS-0976 alone or in combination, in NAFLD patients with advanced fibrosis	Phase 2	Ongoing
NCT02913105	LMB763, dose not revealed, once daily, 12 weeks	Objective: effects of LMB763 with respect to safety, tolerability, and inflammation in patients with NAFLD	Phase 2	Ongoing
NCT02855164	LJN452 or placebo, dose and frequency not revealed, 12 weeks	Objective: effects of different doses of LJN452 with respect to safety, tolerability, and on markers of liver inflammation in patients with NAFLD	Phase 2	Ongoing

OCA: obeticholic acid; T2DM: type 2 diabetes mellitus; NAFLD: nonalcoholic fatty liver disease.

## Additional Points

*Guarantor of the article*. Chunpeng Zhu is the guarantor of the article.
